# WU Polyomavirus in Children with Acute Lower Respiratory Tract Infections, South Korea

**DOI:** 10.3201/eid1311.070872

**Published:** 2007-11

**Authors:** Tae Hee Han, Ju-Young Chung, Ja Wook Koo, Sang Woo Kim, Eung-Soo Hwang

**Affiliations:** * Inje University College of Medicine, Seoul, South Korea; † Seoul National College of Medicine, Seoul, South Korea

**Keywords:** WU polyomavirus, KI polyomavirus, children, acute respiratory tract infection, South Korea, dispatch

## Abstract

In South Korea, WU polyomavirus (WUPyV) was detected in 34 (7%) of 486 children with acute lower respiratory tract infections, 3 (4.2%) of 72 asymptomatic children, and as coinfection with other respiratory viruses in 23 (67.6%) children. Although WUPyV was frequently detected, its clinical role has not been distinguished from that of coinfecting viruses.

The polyomaviruses JC virus and BK virus usually produce asymptomatic infections, but in immunocompromised patients, they can become oncogenic or induce disease (*1*–*3*). In 2007, new polyoma viruses such as WU polyomavirus (WUPyV) and KI polyomavirus (KIPyV) were identified in respiratory specimens from children with acute respiratory tract infections (*4*,*5*). Gaynor et al. (*4*) reported WUPyV prevalence of 3.0% in Australia and 0.6% in the United States. Allander et al. (*5*) reported 1.0% prevalence of KIPyV in nasopharyngeal aspirates from children with mainly respiratory tract diseases. However, the clinical roles of WUPyV and KIPyV during acute respiratory tract infection needed to be clarified because of the high frequency of their codetection with other respiratory viruses (*4*,*5*). The purpose of our study was to determine prevalence of recently identified WUPyV and KIPyV in children who were asymptomatic and children who had acute lower respiratory tract infection.

## The Study

At Sanggyepaik Hospital, Seoul, South Korea, from September 2006 through June 2007, nasopharyngeal aspirates were collected from 558 children <6 years of age: 486 were hospitalized with acute lower respiratory tract infection, and 72 were asymptomatic (those who visited the well-being clinic or were being admitted for elective surgery). Informed consent was obtained from the children’s parents, and the study was approved by the internal review board of Sanggyepaik Hospital.

Viral RNA was extracted from each sample by using a QIAamp Viral RNA Mini Kit (QIAGEN GmbH, Hilden, Germany), and reverse transcription of 0.5 μg of each RNA sample was performed. All nasopharyngeal aspirates from the study population were tested for common respiratory viruses such as human respiratory syncytial virus (hRSV), influenza virus A and B, parainfluenza virus (PIV), and adenovirus by using multiplex reverse transcription–PCR (RT-PCR) (*6*,*7*). The rest of each specimen was then frozen at –80^o^C until tested. RT-PCR assays were performed for rhinovirus (RV), human metapneumovirus (hMPV), human coronavirus (hCoV)–NL63, hCoV-OC43, hCoV-229E, and hCoV HKU-1, as described (*8*–*13*). Positive and negative controls were included in each experiment.

DNA was extracted from the aspirates by using a QIAamp DNA Blood Mini Kit (QIAGEN GmbH). PCR assays were performed to detect human bocavirus (hBoV) by using primers for the nonstructural-1 and nucleoprotein-1 genes, as described (*14*). To detect WUPyV, PCR was performed by using primers AG0044 (5′-TGT TAC AAA TAG CTG CAG GTC AA-3′) and AG0045 (5′-GCT GCA TAA TGG GGA GTA CC-3′); confirmation was performed by using primers AG0048 (5′-TGT TTT TCA AGT ATG TTG CAT CC-3′) and AG0049 (5′-CAC CCA AAA GAC ACT TAA AAG AAA-3′), as described (*4*). KIPyV was detected by nested PCR assays that used primers POLVP1-39F, POLVP1-363R, POLVP1-118F, and POLVP1-324R, as described (*5*). The plasmids containing major capsid protein (VP)–1 region of KIPyV and VP-2 region of WUPyV as positive control were donated by Tobias Allander and David Wang. All PCR products for WUPyV and KIPyV were sequenced to confirm the specificity for each virus. PCR product was examined after electrophoresis on a 1% agarose gel. Amplicon was purified and sequenced in both directions. Nucleotide sequences were aligned by using BioEdit version 7.0 (www.mbio.ncsu.edu/bioedit/bioedit.html) and presented in a topology tree, prepared in MEGA 3.1 (*15*). Using SAS software version 8.02 (SAS Institute, Inc., Cary, NC, USA), we performed the Fisher exact test to compare the proportion of symptomatic WUPyV patients with those who were in the control groups.

Of the 486 children who were hospitalized with acute respiratory tract infections, median age was 9 months (range 1–69 months); of the 72 asymptomatic children, median age was 14 months (range 1–77 months). The age distribution of patients with respiratory tract infections was 220 (45.3%) <12 months, 196 (40.3%) 12–23 months, and 70 (14.4%) 24–69 months. The male:female ratios of the symptomatic children (1.9:1) and asymptomatic children (1.7:1) did not differ significantly. The clinical diagnoses for the 486 symptomatic children were bronchiolitis for 250 children, pneumonia for 201, and croup for 35. Most children in both groups had no additional underlying medical conditions at the time of admission.

Viruses were detected in 407 (83.7%) of the 486 symptomatic children. The most frequently detected agents were hRSV in 101 (20.8%), RV in 91 (18.7%), hBoV in 51 (10.5%), PIV in 48 (9.8%), and hMPV in 34 (7.0%) (Table). WUPyV was found in 34 (7%) and KIPyV in 5 (1%) children in this group. A single virus infection with WUPyV was confirmed for 11 patients (2.2%) and KIPyV for 1 (0.2%). WUPyV was detected along with other viruses in 23 (67.6%) children.

**Table:** Detection of viruses among 486 children with acute lower respiratory tract infection, Seoul, South Korea*

Virus	No. positive (%)
Single virus infection	407 (83.7)
HRSV	101 (20.8)
RV	91 (18.7)
HBoV	51 (10.5)
PIV†	48 (9.9)
hMPV	34 (7.0)
WUPyV	34 (7.0)
AdV	22 (4.5)
HCoV‡	15 (3.0)
Influenza virus	6 (1.2)
KIPyV	5 (1.0)
Coinfection with polyomaviruses and other viruses	27 (5.5)
WUPyV + hRSV	6
WuPyV + RV	5
WUPyV + PIV	5
WUPyV + hMPV	2
KIPyV + PIV	2
KIPyV + hCoV-NL63	1
KIPyV + RV	1
WUPyV + RV + PIV	2
WUPyV + hBoV + PIV	1
WUPyV + hRSV + hBoV	1
WUPyV + hCoV-NL63 + PIV	1

*HRSV, human respiratory syncytial virus; RV, rhinovirus; hBoV, human bocavirus; PIV, parainfluenzavirus; hMPV, human metapneumovirus; WUPyV, WU polyomavirus; AdV, adenovirus; HCoV, human coronavirus; KIPyV, KI polyomavirus. †PIV type 1 in 23 patients, PIV type 2 in 7, PIV type 3 in 10, and PIV type 4 in 8. ‡hCoV-NL63 in 3 patients, hCoV-OC43 in 1, and hCoV-229E in 1.

In the asymptomatic group, KIPyV was not detected, but WUPyV was found in 3 (4.2%) children. Significant difference of prevalence between symptomatic and asymptomatic patients was not noted with WUPyV infection (p = 0.6) but was found with hBoV infection (p = 0.001). Most of the WUPyV infections were detected in May and June (Figure). The clinical symptoms of WUPyV infection in children were similar to those of other viral respiratory tract infections such as hRSV and hBoV. Gastrointestinal symptoms were found in 23.5% (8/34) of WUPyV-positive children. The 31 WUPyV strains detected in symptomatic children and 3 WUPyV strains in asymptomatic children, which were directly sequenced, clustered into 1 VP2 lineage (GenBank accession nos. EF639268–EF639288, EF655818–655825, and EU041602–041606). Our isolates showed 98%–100% nucleotide identity with the VP-2 region of the WUPyV reference strain (S1). Analysis of the KIPyV strains showed the same sequence with the VP-1 region of the KIPyV strain (GenBank accession nos. EF639289, EF655826–655827, and EU041609–041610).

**Figure:**
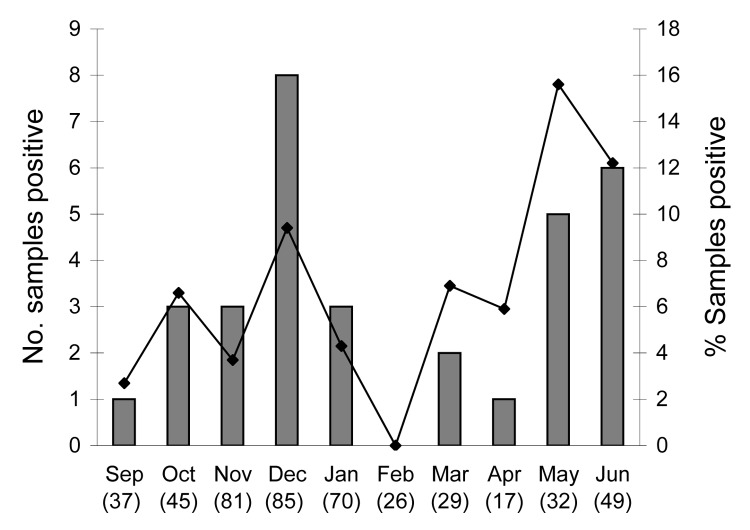
Seasonal distribution of WU polyomavirus (WUPyV) in children hospitalized with acute lower respiratory tract infection, September 2006–June 2007. Total no. WUPyV-positive samples = 34. Number in parentheses after each month is total number of samples tested.

## Conclusions

This prospective study shows that recently identified WUPyVs are prevalent in South Korean children with acute lower respiratory tract infections. Our detection of WUPyV in 34 (7.0%) of 486 children with acute lower respiratory tract infection suggests that the virus is prevalent in South Korea. Our finding of 27 (6.5%) WUPyV-positive samples among 416 patients <24 months of age compared with 7 (10%) positive samples from 70 patients >24 months of age suggests that WUPyV infection may occur more frequently in older children than in younger children; however, studies for latent infection of the virus are needed to confirm. Clinical diagnoses for patients with WUPyV-positive results only included bronchiolitis, tracheobronchitis, pneumonia, and croup. Coinfection of WUPyV with other respiratory viruses, especially hRSV, did not seem to influence the severity of disease, although we did not perform statistical analysis. Although our study tested for more viruses than previous studies and included a control group, whether detection of WUPyV in nasopharyngeal specimens means infection or just transmission in the respiratory tract remains unclear. We performed PCR assays for WUPyV in stool samples from 72 children who had acute gastroenteritis during the same study period, but it was not detected (data not shown).

Our study’s limitations include small population size, short study period, lack of testing for bacterial pathogens, and limitation to hospitalized patients. Our finding of KPyV in 5 (1.0%) of 486 children who had respiratory symptoms and were mostly coinfected with other respiratory viruses is similar to that of a previous study (*5*). In conclusion, WUPyV was frequently found in South Korean children with acute lower respiratory tract infections, but further studies are needed to distinguish the clinical role of this virus from that of other coinfecting respiratory viruses.
